# Rumors and incorrect reports are more deadly than the new coronavirus (SARS-CoV-2)

**DOI:** 10.1186/s13756-020-00738-1

**Published:** 2020-05-19

**Authors:** Rostam Jalali, Masoud Mohammadi

**Affiliations:** Department of Nursing, School of Nursing and Midwifery, Kermanshah University of Medical Sciences, Kermanshah, Iran

In late 2019, the outbreak of a new virus called SARS-CoV-2 in Wuhan, China raised concerns about the global effects of this pandemic [1].

The disease is rapidly progressing, and has affected the whole world [1]. This is concerning as COVID-19 is an acute respiratory disease with a mortality rate of 2% that can be deadly due to high alveolar injury and progressive respiratory failure [1–3].

According to the latest statistic reported on April 17, 2020, more than 2,034,802 infected cases and more than 135,163 cases of death were recorded globally [1–4]. Accurate and correct statistics help a lot in determining the condition of the issue and providing appropriate solutions to control the situation and alleviate the problem.

Nowadays, one of the most effective ways to raise awareness of any issues and raise information about the problems of society, especially diseases and public readiness to contract it, is to use social media, but sometimes these applications, allow inaccurate information and statistics to be spread which can cause hygienic systems to fail.

The outbreak of this pandemic, has resulted in a lot of fear and panic being spread across the world, especially in developing countries, where most of this panic is a result of inaccurate statistics and rumors spread by social networks. The fear mongering and panic that is caused by said social networks spreading inaccurate statistics that leads to an awareness shortage could prove to be more catastrophic than the virus itself in these countries. Since then, the influx of people desperate to get masks and disinfectants has been very noticeable, as some countries have been exposed to masks shortage. There is very little information regarding how to use the masks and who needs access to said masks, as people panic to purchase them without having done any reliable research.

However, if social media is used correctly and the right information for the use of disinfectants, masks and protective equipment is provided, this will not only benefit, but also control the infection and transmission of the SARS-CoV-2 virus significantly.

Controlling the rate of infection caused by the virus, SARS-CoV-2, can be done faster by informing the public, and the fear of not knowing about this virus can be replaced by knowing the methods of controlling the infection and using social networks to provide accurate statistics, which is achievable.

Raising public awareness about the pandemic, can be very effective in reducing the transmission of the disease; Now, if this information is proposed through invalid statistics outside of global health institutions and through social networks, it will not only increase the risk of infection, but may act like a deadly poison that will also disable national healthcare systems. Therefore, it is necessary for hygiene and health policy makers to raise public awareness about these diseases and to transmit statistics through valid channels.

## Data Availability

Datasets are available through the corresponding author upon reasonable request.
